# In Silico Mutational Analysis of Two-Component System Genes Associated with Colistin Resistance in Clinical *Pseudomonas aeruginosa* Isolates from Peshawar

**DOI:** 10.3390/biom16070962

**Published:** 2026-06-29

**Authors:** Bashir Ahmad, Qaisar Ali, Sadiq Azam, Muhammad Asghar, Noor Rehman, Gul-e-Sehra Mujib, Syed Sohail Shah, Jamila Javed, Ibrar Khan, Taj Ali Khan, Taane G. Clark

**Affiliations:** 1 Center of Biotechnology and Microbiology, University of Peshawar, Peshawar 25120, Khyber Pakhtunkhwa, Pakistan; bashir.biotech1@gmail.com (B.A.); qaisar_researcher@uop.edu.pk (Q.A.); gulesehramujib@uop.edu.pk (G.-e.-S.M.); sohail.syed@uop.edu.pk (S.S.S.); ibrarkhan1984@uop.edu.pk (I.K.); 2 Department of Pathology, Khyber Medical College, Peshawar 25000, Khyber Pakhtunkhwa, Pakistan; drazghar@gmail.com (M.A.); noorbangash61@gmail.com (N.R.); 3 Institute of Biotechnology Genetic Engineering, The University of Agriculture, Peshawar 25130, Khyber Pakhtunkhwa, Pakistan; jamilajaved21@gmail.com; 4 Institute of Pathology and Diagnostic Medicine, Khyber Medical University, Peshawar 25120, Khyber Pakhtunkhwa, Pakistan; tajalikhan.ibms@kmu.edu.pk; 5 Faculty of Infectious and Tropical Diseases, London School of Hygiene and Tropical Medicine, London WC1E 7HT, UK; 6 Faculty of Epidemiology and Population Health, London School of Hygiene and Tropical Medicine, London WC1E 7HT, UK

**Keywords:** *Pseudomonas aeruginosa*, colistin resistance, two-component system, molecular docking, ANP, *mcr*-1

## Abstract

*Pseudomonas aeruginosa* is an opportunistic pathogen causing healthcare-associated infections. Colistin is a last-resort antibiotic for multidrug-resistant Gram-negative bacteria. Resistance arises through mutations in two-component systems (TCS) regulating the *arn* operon. Data on colistin resistance in *P. aeruginosa* from Pakistan remain limited. A total of 3189 clinical samples (urine, blood, sputum, pus, wound swabs) were cultured. *P. aeruginosa* was identified by Gram staining, biochemical tests (catalase, oxidase, API 20E), and *oprL* gene amplification. Antibiotic susceptibility was determined by disk diffusion and MIC strips. Resistance genes (*PhoP*, *PhoQ*, *PmrA*, *PmrB*, *mcr*-1, *oprD*) were detected by PCR and Sanger sequencing. Wild-type protein structures were retrieved from PDB; mutant structures were predicted using AlphaFold3. ANP (phosphoaminophosphonic acid-adenylate ester) was docked using MOE 2019.0102. Of 3189 samples, 384 (12.0%) yielded *P. aeruginosa*. Wound/pus (38.0%) and surgical wards (30.0%) were the predominant sources. Colistin and polymyxin B showed 99.0% susceptibility (MIC_50_/MIC_90_ = 1 µg/mL). High resistance was observed for Piperacillin–Tazobactam (96.4%), Aztreonam (70.6%), and Gentamicin (64.2%). *oprD* was the most prevalent gene (87.5%), followed by *PmrB* (54.0%), *PhoQ* (44.0%), *PhoP* (36.0%), *PmrA* (18.0%), and *mcr*-1 (8.0%). Docking revealed the strongest binding in wild-type PhoQ (1ID0; −12.0 kcal/mol, LYS392), wild-type PmrB (2JSO; −9.8 kcal/mol, ASP37), and wild-type PhoP (2PKX; −9.1 kcal/mol, LYS87/ARG111). Mutant proteins showed reduced binding affinities and dispersed interaction networks. Mutant PhoP formed 16 contacts (strongest −4.3 kcal/mol) versus wild-type PhoP with 13 contacts (−9.1 kcal/mol). Colistin remains highly effective against *P. aeruginosa* in this setting (99.0% susceptibility). The presence of *mcr*-1 (8.0%) and high *oprD* prevalence (87.5%) require continued surveillance. Mutations in TCS proteins reduce ANP binding affinity and alter interaction specificity, suggesting that ATP-competitive inhibitors targeting these kinases merit further investigation and experimental validation.

## 1. Introduction

*Pseudomonas aeruginosa* is a ubiquitous Gram-negative bacillus commonly found in soil, water, and plant-associated environments. Its metabolic adaptability and natural resistance to multiple antimicrobial agents enable survival in diverse conditions, making it an important opportunistic pathogen [1]. It frequently infects individuals with compromised immune systems, including patients with severe burns, cancer, or cystic fibrosis. *P. aeruginosa* commonly colonizes the respiratory tract, urinary tract, and damaged tissues, leading to infections such as pneumonia, urinary tract infections (UTIs), and wound infections [2]. It is a major cause of healthcare-associated infections and represents a persistent problem in hospital environments [3]. The organism also forms biofilms on biotic and abiotic surfaces that increase tolerance to antimicrobial agents and disinfectants and facilitate its persistence on medical devices and within clinical settings [4]. The rise in multidrug-resistant (MDR) *P. aeruginosa* represents a critical global health threat [5]. *P. aeruginosa* is a multidrug-resistant opportunistic pathogen that causes acute or chronic infections in immunocompromised individuals with conditions such as cystic fibrosis, cancer, burns, sepsis, and ventilator-associated pneumonia, including those caused by COVID-19 [6]. The overuse and misuse of antibiotics have driven the selection of resistant strains, compromised the efficacy of conventional therapies, and led to difficult-to-treat infections [7]. This trend underscores the urgent need for novel therapeutic approaches and infection control strategies to combat this formidable pathogen. In *P. aeruginosa*, colistin resistance arises from mutations in two-component systems (TCS) that regulate the *arn* operon, mediating lipopolysaccharide (LPS) modifications and enhancing polymyxin resistance [8]. TCS, composed of a sensor histidine kinase and a response regulator, is central to environmental sensing and adaptive responses [9]. In resistant strains, TCS genes such as *PhoPQ*, *PmrAB*, and *CprRS* control *arn*-mediated LPS modifications, diminishing colistin binding and efficacy [10]. Mutations in *CprRS* suppress *arn* operon activation, whereas alterations in *PhoPQ* and *PmrAB* upregulate its expression, increasing cell surface hydrophobicity and reducing antibiotic binding [11]. Mutations in other TCS further enhance resistance by activating alternative regulatory pathways. Upregulation of the *arn* operon promotes incorporation of 4-amino-4-deoxy-L-arabinose (Ara4N) into lipid A, a critical LPS component, thereby diminishing colistin binding and conferring resistance [12]. Colistin resistance in *P. aeruginosa* varies by region and is poorly reported in Pakistan, where unregulated antibiotic use has created antimicrobial resistance, especially in tertiary care hospitals in Peshawar. The present study is designed to characterize *P. aeruginosa* isolates from Khyber Teaching Hospital, Peshawar, using phenotypic assays, molecular techniques, and in silico analyses, with the aim of explaining the structural basis of colistin resistance and identifying potential therapeutic targets to inform infection control and AMR management.

## 2. Materials and Methods

### 2.1. Sample Collection, Bacterial Isolation, and Identification

The current cross-sectional study was conducted from January to December 2022 at the Pathology Laboratory, Khyber Teaching Hospital (KTH), Peshawar, and the Genomics Laboratory, Center for Biotechnology and Microbiology, University of Peshawar. Ethical approval was obtained from the Institutional Review Board (IRB) of KTH (Approval No. 124/DME/KMC; 11 February 2021). All procedures complied with the Declaration of Helsinki, and written informed consent was obtained from all participants or their legal guardians prior to sample collection. A total of 3189 clinical samples (urine, blood, sputum, pus, and wounds) were collected in sterile containers, swabs, bottles, and syringes from inpatient (IPD) and outpatient (OPD) departments. All bacterial isolates were obtained from distinct individual patients, with each patient contributing only one clinical sample. No replicate samples were included in the study. Upon informed consent and medical history, clinical samples were transported directly to the pathology laboratory, KTH. Variables, including age, gender, and sample source, were recorded for comparative analysis. Bacterial infections were included, while fungal and viral infections were excluded. Samples were inoculated onto blood agar and MacConkey agar (Oxoid, Basingstoke, UK) and incubated overnight at 37 °C. Bacterial colonies were sub-cultured to obtain pure isolates [13]. Colonies were examined for size, shape, color, and texture. Gram staining was performed to assess the Gram status of the species. Phenotypic identification was performed by colony morphology, Gram staining, and the API-20-E system (BioMérieux, Marcy-l’Étoile, France), with results interpreted per the manufacturer’s identification key. Molecular confirmation of the isolates was carried out by PCR amplification of the *oprL* gene using specific primers under optimized conditions (Supplementary Table S1). The resulting amplicons were identified on a 1.5% agarose gel and visualized with a Bio-Rad Molecular Imager^®^ Gel Doc™ system (Bio-Rad Laboratories, Inc., Hercules, CA, USA). Confirmed bacterial isolates were preserved at −20 °C in Tryptic Soy Broth (TSB) supplemented with 16% glycerol [14,15].

### 2.2. Antibiotic Susceptibility Testing and DNA Extraction

Antibiotic susceptibility was determined using the disk diffusion (Kirby–Bauer) method. Bacterial cultures were inoculated onto Mueller–Hinton agar plates. Antibiotic discs were placed on the agar surface with sterile forceps, followed by incubation at 37 °C for 24 hours. Zones of inhibition were measured and interpreted as susceptible, intermediate, or resistant according to Clinical and Laboratory Standards Institute (CLSI) 2023 guidelines [16]. Minimum Inhibitory Concentration (MIC) strips were used to determine the lowest antibiotic concentration inhibiting bacterial growth. The MIC was determined by observing the point of visible growth inhibition along the strip [17]. The following antibiotics were tested: Levofloxacin, Ceftazidime, Cefoperazone–Sulbactam, Piperacillin–Tazobactam, Cefepime, Meropenem, Amikacin, Imipenem, Gentamicin, Aztreonam, Tobramycin, Ciprofloxacin, Fosfomycin, Polymyxin B, Doripenem, and Colistin. Bacterial DNA was extracted using a commercial kit (QIAamp DNA Mini Kit; QIAGEN, Hilden, Germany). DNA purity and concentration were assessed by spectrophotometry (OD260/280 ratio) and agarose gel electrophoresis. Purified DNA was stored at −20 °C for further analysis [18].

### 2.3. Molecular Characterization and Mutational Analysis of Resistance Genes

Specific primers were designed for the amplification of Colistin resistance-associated genes (*PhoP*, *PhoQ*, *PmrA*, *PmrB*, *mcr*-1, and *oprD*) in *P. aeruginosa* based on the antibiotic resistance pattern. Primer specificity was validated in silico by BLAST (v2.17.0) analysis against the *P. aeruginosa* PAO1 reference genome (NCBI accession: NC_002516.2). The reference sequences used for primer design were: *PhoP* (PA1179), *PhoQ* (PA1180), *PmrA* (PA4776), *PmrB* (PA4777), and *oprD* (PA0958). For *mcr*-1, plasmid-mediated sequences available in GenBank were used as references. Amplified PCR products were analyzed by gel electrophoresis alongside a DNA ladder and positive and negative controls. The gel was visualized using gel documentation [19]. Amplified colistin resistance genes were sent to the genomics laboratory at Khyber Medical University (KMU), Hayatabad, Peshawar, for Sanger sequencing. Sequence files were analyzed using bioinformatics tools, including BLAST (v2.17.0), BioEdit (v7.7), and CLUSTALW (v2.1), for mutational analysis [20].

### 2.4. In Silico Mutation and Molecular Docking

To explore therapeutic strategies, 3D structures of each Mutant protein (PhoP, PhoQ, PmrB, and mcr-1) were created using AlphaFold3. Wild structure for each protein was retrieved from the PDB database. The ligand, phosphoaminophosphonic acid-adenylate ester (ANP, C_10_H_17_N_6_O_12_P_3_; molecular weight 506.196 g/mol), was docked individually against all four wild-types and corresponding mutant protein targets to compare the changes in binding affinity and interaction patterns. Molecular docking was performed using the Molecular Operating Environment (MOE 2019.0102). The protein structures were prepared by protonation, energy minimization, and removal of water molecules. The ligand structure was energy-minimized using the MMFF94x force field. Docking simulations were conducted using the Triangle Matcher placement method and the London dG scoring function. The top-scoring poses were refined using the Forcefield refinement method, and the best conformation was selected based on binding affinity and interaction profiles (hydrogen bonds, hydrophobic interactions, and electrostatic forces). Docking results for wild-type and mutant proteins were compared to assess the impact of mutations on ligand binding [21].

### 2.5. Statistical Analysis

Statistical analyses were performed using SPSS software (v26.0). Categorical variables (gender, age groups, specimen source, hospital department, antibiotic susceptibility, and gene presence/absence) were analyzed using the chi-square (χ^2^) test. For docking studies, only single representative docking scores were generated per protein; therefore, statistical comparisons between wild-type and mutant groups were not performed. A *p*-value < 0.05 was considered statistically significant for all other analyses [22].

## 3. Results

### 3.1. Bacterial Isolation and Identification

A total of 3189 clinical specimens cultured on blood agar, MacConkey agar, and cetrimide agar, 384 (12.0%) yielded *P. aeruginosa* after 24 h of incubation at 37 °C. On MacConkey agar, colonies appeared pale or colorless, consistent with non-lactose fermentation (Figure S1A). On cetrimide agar, colonies displayed greenish-blue pigmentation and yellow-green fluorescence under ultraviolet light (Figure S1B). Gram staining revealed pink, rod-shaped, Gram-negative bacilli arranged singly or in short chains (Figure S1C). Catalase and oxidase tests were positive (Figure S1E,F). The API 20E system confirmed *P. aeruginosa* isolate number 2212004, with positive reactions for arginine dihydrolase (ADH), lysine decarboxylase (LDC), hydrogen sulfide (H_2_S), indole production (IND), glucose fermentation, gelatin hydrolysis, mannitol fermentation, saccharose fermentation, and melibiose fermentation. Negative reactions were observed for ortho-nitrophenyl-β-D-galactopyranoside (ONPG), citrate utilization (CIT), Voges–Proskauer (VP), urease (URE), maltose, arabinoside, and amylose (Figure S1D). Molecular identification through amplification of the *oprL* gene produced a 504 bp product, confirming *P. aeruginosa* (Figure S2).

### 3.2. Demographic and Epidemiological Data

#### Distribution of Clinical Samples and *P. aeruginosa* Isolates

A total of 3189 clinical samples were analyzed, of which 384 (12.0%) were positive for *P. aeruginosa*. Among gender groups, a slightly higher prevalence was observed in males (12.9%) compared to females (10.8%); however, this difference was not statistically significant (χ^2^ = 3.14, *p* = 0.076). Age-wise distribution showed a gradual increase in positivity with advancing age, ranging from 8.5% in individuals ≤10 years to 13.0% in individuals >60 years, but the association was also not statistically significant (Table 1, χ^2^ = 3.52, df = 4, *p* = 0.474).

### 3.3. Source-Wise Distribution of P. aeruginosa Isolates

Out of 384 confirmed *P. aeruginosa* isolates, the highest proportion was obtained from wound/pus samples (38.0%), followed by urine (27.1%) and sputum (18.0%), while blood (9.9%) and other specimen types (7.0%) contributed fewer isolates. The distribution of isolates across different specimen sources was found to be statistically significant (Table 2, χ^2^ = 89.6, df = 4, *p* < 0.001), indicating a strong association between the type of clinical specimen and the occurrence of *P. aeruginosa*.

### 3.4. Distribution of Pseudomonas aeruginosa Isolates by Hospital Department

Among the 384 *P. aeruginosa* isolates, the highest frequency was observed in surgical wards (30.0%), followed by the intensive care unit (25.0%) and burn unit (18.0%), while lower proportions were recorded in medical wards (15.1%), outpatient department (8.1%), and pediatric unit (3.9%). The distribution was statistically significant (Table 3, χ^2^ = 74.8, df = 5, *p* < 0.001), indicating a strong association between hospital department and the occurrence of *P. aeruginosa*, with a higher burden in critical and high-risk care settings.

### 3.5. Antibiotic Susceptibility Profile of Pseudomonas aeruginosa

The antibiotic susceptibility analysis of the 384 *P. aeruginosa* isolates demonstrated significant variability across tested agents. The highest sensitivity was observed for polymyxins, with both colistin and polymyxin B showing 99.0% susceptibility, followed by Carbapenems including Imipenem (94.8%), Meropenem (91.6%), and Doripenem (90.0%), as well as amikacin (92.9%). In contrast, high resistance rates were noted against Piperacillin–tazobactam (96.4%), Cefoperazone–sulbactam (83.1%), Aztreonam (70.6%), and Aminoglycosides such as Gentamicin (64.2%) and Tobramycin (60.3%). Moderate susceptibility was observed for Ceftazidime, Cefepime, and Fluoroquinolones. The overall distribution of susceptibility and resistance patterns was statistically significant (Table 4, χ^2^ = 1652.3, df = 14, *p* < 0.001), indicating a strong variation in antibiotic effectiveness against *P. aeruginosa* isolates.

### 3.6. Minimum Inhibitory Concentration (MIC) Profile of Pseudomonas aeruginosa

The MIC analysis of selected antibiotics against 384 *P. aeruginosa* isolates demonstrated strong efficacy of polymyxins, with both colistin and polymyxin B showing low MIC_50_ and MIC_90_ values (1 µg/mL) within the susceptible range. Carbapenems exhibited variable activity, where meropenem showed an MIC_50_ of 1 µg/mL and an MIC_90_ of 8 µg/mL, while imipenem displayed slightly higher MIC values (Table 5, MIC_50_ = 2 µg/mL, MIC_90_ = 16 µg/mL), indicating emerging resistance.

### 3.7. Association of Resistance Genes with Phenotypic Antibiotic Resistance in P. aeruginosa

The comparative analysis of selected *P. aeruginosa* isolates demonstrated a clear association between the presence of resistance-associated genes and corresponding phenotypic resistance profiles. Isolates harboring multiple regulatory genes such as *PhoP*, *PhoQ*, *PmrA*, and *PmrB*, along with *mcr*-1, exhibited broad-spectrum resistance to polymyxins, carbapenems, cephalosporins, and fluoroquinolones. Isolates containing *mcr*-1 showed resistance to colistin and polymyxin B, while the presence of *oprD* was linked to carbapenem resistance. Isolates with a higher number of resistance genes displayed more extensive multidrug-resistant phenotypes, highlighting the genetic basis of antimicrobial resistance in *P. aeruginosa* (Table 6).

### 3.8. Gene–Antibiotic Resistance Association Matrix in Pseudomonas aeruginosa

The gene–antibiotic resistance association matrix highlights distinct relationships between resistance determinants and phenotypic antibiotic response in *P. aeruginosa*. Strong associations (+++) were observed between *mcr*-1 and polymyxin resistance (colistin and polymyxin B), as well as between *oprD* and carbapenem resistance (meropenem and imipenem). The *PmrB* gene demonstrated a broad impact, showing consistent association with resistance to multiple antibiotic classes. Moderate associations (+) were noted for *PhoP*, *PhoQ*, and *PmrA* with Polymyxins, Cephalosporins, and Fluoroquinolones, while variable associations (±) were observed across several antibiotics, indicating heterogeneity in resistance expression. The absence of an association (−) between *oprD* and polymyxins and certain other antibiotics further supports its specific role in carbapenem resistance (Table 7 and Table 8).

### 3.9. Classification of Multidrug-Resistant, Extensively Drug-Resistant, and Pan-Drug-Resistant Isolates

The 384 *P. aeruginosa* isolates were classified according to the international consensus definitions proposed by Magiorakos et al. (2012) [26], which categorize resistance based on the number of antimicrobial categories to which isolates demonstrate non-susceptibility. Seven antimicrobial categories were evaluated: Polymyxins (Colistin, Polymyxin B), carbapenems (Imipenem, Meropenem, Doripenem), Cephalosporins (Ceftazidime, Cefepime), Penicillin (Piperacillin–Tazobactam, Cefoperazone–Sulbactam), Aminoglycosides (Amikacin, Gentamicin, Tobramycin), Fluoroquinolones (Ciprofloxacin, Levofloxacin), and Monobactams (Aztreonam).

### 3.10. Mutational Analysis of Genes Involved in Colistin Resistance

All 384 *P. aeruginosa* isolates carried the intrinsic chromosomal genes *PhoP* (PA1179), *PhoQ* (PA1180), *PmrA* (PA4776), *PmrB* (PA4777), and oprD (PA0958), as expected from the *P. aeruginosa* PAO1 reference genome. Sanger sequencing of these genes, along with the plasmid-mediated *mcr*-1 gene, identified a total of 24 non-synonymous amino acid substitutions across the six genes. The highest mutational load was observed in *PhoQ* (*n* = 9), followed by *PmrA* (*n* = 5) and *PmrB* (*n* = 5). *PhoP* and *mcr*-1 each carried two mutations, while *oprD* showed a single substitution. The frequency of isolates harboring mutations varied across genes: *oprD* (87.5%), *PmrB* (54.0%), *PhoQ* (44.0%), *PhoP* (36.0%), *PmrA* (18.0%), and *mcr*-1 (8.0%). The distribution of mutations was statistically significant (χ^2^ = 412.6, df = 5, *p* < 0.001). In *PmrA*, five substitutions were detected (p.S73, p.W145C, p.T167N, p.S187C, p.25W). *PmrB* harbored five variants (p.S8X, p.G121D, p.Q196L, p.E400G, p.E461X). The *PhoQ* gene exhibited nine amino acid changes (p.C28W, p.R42G, p.R51G, p.S63A, p.A88G, p.R173C, p.A192G, p.238W, p.A260T). *PhoP* contained p.V208F and p.C260Y, while *oprD* showed p.D236G. The plasmid-mediated *mcr*-1 gene carried two variants (p.S30, p.C54F). Details are provided in Supplementary Table S2.

### 3.11. Molecular Docking Analysis

Molecular docking studies were performed using MOE 2019.0102 to investigate the binding interactions of ANP (phosphoaminophosphonic acid-adenylate ester) with selected target proteins. Out of all proteins, binding energy analysis identified Wild-PhoQ (PDB ID: 1ID0) as the strongest binding system, exhibiting a binding energy of −12.0 kcal/mol, primarily mediated by the amino acid residue at LYS392. High-affinity interactions were also observed in Wild-PmrB (PDB ID: 2JSO; ASP37: −9.8 kcal/mol) and Wild-PhoP (PDB ID: 2PKX; LYS87/ARG111: −9.1 kcal/mol), indicating stable ligand recognition in these systems. In contrast, mutant protein systems showed comparatively lower binding energies but varying interaction complexities. Interaction profiling revealed that Mut PhoP formed the highest number of ligands–protein contacts (16 interactions), suggesting a broad and distributed binding interface, whereas 2JSO displayed a highly focused single-residue interaction pattern. Across all protein systems, charged amino acids-particularly LYS, ARG, GLU, and ASP-were consistently involved in ligand stabilization, highlighting the dominant role of electrostatic interactions in ANP binding. Hydrogen bonding (H-donor and H-acceptor interactions) served as the primary stabilizing force, while π–H, π–cation, and π–π interactions contributed secondary structural stabilization in selected systems (Table 9 and Table 10).

### 3.12. Molecular Docking Analysis of Wild and Mutant PhoP with ANP (Phosphoaminophosphonic Acid-Adenylate Ester)

Molecular docking analysis was performed to evaluate the binding interaction of ANP (phosphoaminophosphonic acid-adenylate ester), a non-hydrolysable ATP analogue, with both wild-type and mutant PhoP transcriptional regulator proteins. In the mutant PhoP structure, ANP exhibited stable binding within the active pocket, forming multiple hydrogen-bond interactions primarily with key residues, including ARG179, SER135, ASP62, SER88, and GLN183. The π–H interaction with PHE89 further contributed to ligand stabilization. The interaction distances ranged from 2.75 to 3.48 Å, with binding energies between −0.8 and −4.3 kcal/mol, indicating moderate but distributed binding affinity across several active-site residues. On the other hand, docking of ANP with the wild-type PhoP (2PKX) revealed a comparatively stronger and more electrostatically driven interaction network. The ligand formed multiple ionic interactions, predominantly with positively charged residues, such as LYS87 and ARG111, alongside hydrogen bonding with THR100, GLU107, and HIS104. In particular, strong ionic interactions (up to −9.0 kcal/mol) and shorter bond distances (as low as 2.48 Å) suggest a more stable and tightly coordinated binding environment in the wild-type protein. 2D and 3D diagrams of both wild and mutant PhoP proteins are shown in Supplementary Figures S3 and S4 respectively. The detailed data are presented in Supplementary Table S3.

### 3.13. Molecular Docking Analysis of mcr-1 Mutant and Wild-Type Protein with ANP (Phosphoaminophosphonic Acid-Adenylate Ester)

Molecular docking of ANP (phosphoaminophosphonic acid–adenylate ester) with the mutant mcr-1 protein revealed a binding mode predominantly stabilized by hydrogen bonding interactions. The ligand formed repeated contacts with MET47, exhibiting interaction energies up to −4.0 kcal/mol, along with additional interactions involving ALA15, THR79, and GLY72. This indicates a binding pattern driven by a limited number of polar and backbone residues with moderate energetic contributions. In contrast, docking with the wild-type mcr-1 protein (PDB ID: 3PBT) demonstrated a more diverse interaction network. ANP established hydrogen bonds with ILE347, ASN351, THR487, and SER294, along with π–H interactions involving ALA488 and ARG489. These interactions showed shorter bond distances (2.60–3.19 Å) and stable binding energies (up to −3.4 kcal/mol), reflecting a more favorable and coordinated binding environment. 2D and 3D diagrams of both wild and mutant mcr-1 proteins are shown in Supplementary Figures S5 and S6 respectively. The detailed data are presented in Supplementary Table S4.

### 3.14. Molecular Docking Analysis of PhoQ (Mutant and Wild-Type) with ANP (Phosphoaminophosphonic Acid-Adenylate Ester)

Molecular docking of ANP with the mutant PhoQ protein revealed a binding profile primarily stabilized through hydrogen bonding interactions. The ligand formed a strong interaction with GLU271 (−6.8 kcal/mol), indicating a key anchoring role in the binding pocket. Additional interactions with ALA279, GLN281, LEU278, and HIS286 contributed to ligand stabilization through both donor and acceptor hydrogen bonds, with interaction distances ranging from 2.74 to 3.35 Å. Overall, the mutant PhoQ exhibited a relatively compact interaction network dominated by a limited number of active-site residues. In contrast, docking of ANP with the wild-type PhoQ (1ID0 transferase complex) demonstrated a more extensive and energetically favorable interaction pattern. The ligand engaged multiple residues, including GLN442, ASN385, ASN389, ASP430, VAL444, LYS392, TYR393, ARG439, ARG434, and LEU446, through a combination of hydrogen bonding, ionic interactions, and π-mediated contacts. Notably, LYS392 exhibited a highly strong interaction energy (−12.0 kcal/mol), highlighting a key stabilizing role in ligand binding. Additional π–H and π–cation interactions involving ARG431 and ARG434 further enhanced binding stability. Comparative analysis indicates that the wild-type PhoQ displays a broader and more stable interaction network with ANP, characterized by diverse interaction types and stronger electrostatic contributions, whereas the mutant PhoQ shows a reduced interaction spectrum with fewer residues and comparatively weaker overall stabilization. These findings suggest that mutations in PhoQ may alter the conformational architecture of the active site, potentially affecting ligand binding affinity and downstream regulatory signaling. 2D and 3D diagrams of both wild and mutant PhoQ proteins are shown in Supplementary Figures S7 and S8 respectively. The detailed data are presented in Supplementary Table S5.

### 3.15. Molecular Docking Analysis of PmrB (Mutant and Wild-Type) with ANP (Phosphoaminophosphonic Acid-Adenylate Ester)

Molecular docking of ANP with the mutant PmrB protein revealed a moderately stable interaction profile characterized by a combination of hydrogen bonding, π–H, and π–π interactions. The ligand interacted with key residues including ASN351, ASN355, GLY415, ARG358, and GLY413, forming both donor and acceptor hydrogen bonds with interaction distances ranging from 2.65 to 3.38 Å and energies between −0.6 and −2.0 kcal/mol. Additional stabilization was provided by π–H interaction with ASN355 and a π–π interaction with TYR359, suggesting involvement of aromatic residues in ligand accommodation. Overall, the mutant PmrB displayed a distributed interaction network with moderate binding strength. In contrast, the wild-type PmrB (2JSO signaling protein) exhibited a highly focused and strong interaction with ANP, primarily through a single but energetically significant hydrogen bond with ASP37 (−9.8 kcal/mol) at a short distance of 2.87 Å. This strong interaction indicates a well-defined and high-affinity binding site in the native protein. 2D and 3D diagrams of both wild and mutant PmrB proteins are shown in Supplementary Figures S9 and S10 respectively. The detailed data is presented in Supplementary Table S6.

## 4. Discussion

The increasing global burden of antimicrobial resistance in *Pseudomonas aeruginosa* has made this pathogen a major concern for public health surveillance worldwide. Epidemiological studies have reported that *P. aeruginosa* isolated from European populations exhibits a combined resistance rate of 12.9%, and it is one of the top-listed pathogens causing hospital-acquired infections, accounting for approximately 9% of nosocomial infections in European hospitals and 7.1% in the United States [5]. In China, data from the China Antimicrobial Surveillance Network (CHINET) identified *P. aeruginosa* as the fourth most common nosocomial pathogen, accounting for 7.96% of clinically isolated pathogenic strains. The widespread use of antibiotics and the slow development of effective antimicrobials present daunting challenges and necessitate new therapeutic strategies to combat infections caused by multidrug-resistant strains [5]. Colistin, a polymyxin antibiotic, has been revived as a last-resort therapeutic option for infections caused by multidrug-resistant (MDR) and extensively drug-resistant (XDR) Gram-negative bacteria, including *P. aeruginosa* [27]. However, the emergence of colistin resistance threatens the clinical utility of this agent. Resistance to colistin in *P. aeruginosa* is primarily mediated by chromosomal mutations in two-component regulatory systems (TCS), namely *PhoPQ* and *PmrAB*, which govern the *arn* operon. Activation of these systems leads to the covalent addition of 4-amino-4-deoxy-L-arabinose (L-Ara4N) to the lipid A moiety of lipopolysaccharide (LPS), reducing the negative charge of the outer membrane and consequently diminishing colistin binding affinity. Although plasmid-mediated *mcr* genes are a major concern in Enterobacteriaceae, their role in *P. aeruginosa* remains less defined, though emerging reports indicate their sporadic presence [28].

The present study was designed to characterize colistin resistance in clinical *P. aeruginosa* isolates from Khyber Teaching Hospital, Peshawar, using phenotypic susceptibility testing, molecular detection of resistance genes, and in silico mutational and docking analyses to elucidate the structural basis of resistance and identify potential therapeutic targets. The isolation rate of *P. aeruginosa* in the present study was 12.0% (384/3189). These findings are consistent with a recent report by Ali et al. (2025) from Khyber Pakhtunkhwa, who identified *P. aeruginosa* in 107 clinical samples, although no overall prevalence percentage was reported [28]. In contrast, a markedly higher prevalence of 72% (36/50) was reported by Ijaz et al. (2024) in wound samples from Lahore hospitals [29].

The present study found no significant difference in the prevalence of *P. aeruginosa* between male (12.9%) and female (10.8%) patients (*p* = 0.076). This finding is consistent with that of Iqbal et al. (2023), who reported no significant sex-based variation in *P. aeruginosa* occurrence [30]. Similarly, Ejaz (2022) [31] also reported no significant gender-related difference in postoperative wound infections caused by *P. aeruginosa*. The absence of a sex predilection suggests that exposure risk and host susceptibility factors for this opportunistic pathogen are comparable in males and females.

Analysis of specimen sources revealed that wound/pus samples yielded the highest proportion of *P. aeruginosa* (38.0%), followed by urine (27.1%) and sputum (18.0%). This distribution was statistically significant (χ^2^ = 89.6, *p* < 0.001). Khazaal et al. reported a similar pattern in Peshawar, where pus specimens contributed 40% of *P. aeruginosa* isolates, followed by urine (18%) and sputum (12.6%). The predominance of wound isolates is consistent with the known pathogenicity of *P. aeruginosa* in damaged skin and soft tissues, where its ability to produce elastase, exotoxin A, and biofilm facilitates tissue destruction and persistent colonization [32]. The departmental distribution showed the highest isolation rates in surgical wards (30.0%), ICU (25.0%), and burn unit (18.0%), with statistically significant variation across departments (χ^2^ = 74.8, *p* < 0.001). This pattern reflects the higher burden of *P. aeruginosa* in high-risk, critically ill patient populations. Li et al. (2021) reported that MDR *P. aeruginosa* isolates in Chinese hospitals were predominantly distributed in comprehensive ICUs and burn departments [33].

The antibiotic susceptibility profile of *P. aeruginosa* demonstrated preserved activity of polymyxins, with 99.0% susceptibility to colistin and polymyxin B (MIC_50_/MIC_90_ = 1 µg/mL). In contrast to reports of high colistin resistance in Pakistan, the low resistance rate (1.0%) observed here may reflect restricted clinical use and reduced selective pressure. Carbapenems also retained high efficacy, with susceptibilities of 94.8% for imipenem and 91.6% for meropenem. These findings differ from regional studies reporting widespread carbapenem resistance and carbapenemase production, suggesting geographic variation in antimicrobial resistance patterns and differences in antibiotic exposure among patient populations [34,35]. The molecular analysis revealed a high prevalence of *oprD* (87.5%), followed by *PmrB* (54.0%) and *PhoQ* (44.0%), whereas *mcr*-1 was detected in only 8.0% of isolates. The low frequency of *mcr*-1 is consistent with the high phenotypic susceptibility to colistin observed in this study. Strong associations were observed between *mcr*-1 and polymyxin resistance, and between *oprD* and carbapenem resistance, highlighting their roles in antimicrobial resistance. Isolates carrying multiple regulatory genes (*PhoP*, *PhoQ*, *PmrA*, and *PmrB*) exhibited broader resistance profiles, reflecting the contribution of two-component regulatory systems to lipopolysaccharide modification and adaptive resistance. The significant distribution of resistance genes (χ^2^ = 412.6, *p* < 0.001) supports their association with resistance phenotypes. However, the incomplete correlation between gene presence and phenotypic resistance suggests that resistance is influenced not only by gene carriage but also by regulatory and environmental factors affecting gene expression [36].

Molecular docking analysis revealed that ANP (phosphoaminophosphonic acid-adenylate ester), a non-hydrolysable ATP analogue, binds differentially to wild-type and mutant variants of colistin resistance-associated proteins (PhoP, PhoQ, PmrB, and mcr-1) in *P. aeruginosa*. The highest binding affinity was observed for the wild-type transferase complex (1ID0; −12.0 kcal/mol) and wild-type signaling protein (2JSO; −9.8 kcal/mol), with LYS392 and ASP37 serving as critical anchoring residues, respectively. These findings are consistent with a previous report where ANP docking to the ATP-binding site of a helicase yielded a binding energy of −13.03 kcal/mol. The strong electrostatic contribution from basic residues (LYS, ARG) and acidic residues (GLU, ASP) observed in the present study aligns with the established role of charged amino acids in stabilizing ANP phosphate groups through ionic and hydrogen-bond interactions. Comparative analysis demonstrated that wild-type proteins consistently exhibited stronger binding affinities and more focused interaction networks compared to their mutant counterparts. For instance, wild-type PhoQ (1ID0) formed 14 interactions with diverse residue types (hydrogen bonds, π–H, π–cation), whereas mutant PhoQ formed only six interactions, primarily hydrogen bonds with reduced energetic contribution. Similarly, wild-type PmrB (2JSO) relied on a single high-affinity hydrogen bond with ASP37 (−9.8 kcal/mol), while mutant PmrB displayed a dispersed interaction network involving eight residues with substantially weaker binding energies (−0.6 to −2.0 kcal/mol). This pattern—reduced binding affinity and altered interaction specificity in mutants—is consistent with previous in silico studies of protein–DNA and protein–protein interactions, where single amino acid substitutions disrupted binding pocket architecture and reduced stabilization energy. The mutant PhoP protein formed the highest number of ligand–protein contacts (16 interactions), yet the strongest individual interaction (−4.3 kcal/mol with ARG179) was substantially weaker than the strongest interaction in wild-type PhoP (2PKX; −9.1 kcal/mol with LYS87/ARG111). This inverse relationship between interaction count and binding strength suggests that mutations may induce conformational changes that broaden the binding interface but reduce specific electrostatic complementarity [37]. The wild-type mcr1 protein (3PBT) exhibited a broader interaction landscape with ANP, including hydrogen bonds with ILE347, ASN351, THR487, and SER294, supplemented by π–H contacts with ALA488 and ARG489. In contrast, the mutant MCR-1 protein showed a constrained interaction pattern centered on MET47, with a maximum binding energy of −4.0 kcal/mol. The lower interaction diversity in the mutant suggests that the mutation may alter the structural organization of the binding pocket, potentially affecting ligand accommodation.

ANP (adenylyl-imidodiphosphate) was selected as the docking ligand because it is a non-hydrolysable ATP analogue commonly used to investigate ATP-binding pockets and kinase–ligand interactions. Since PhoQ and PmrB are ATP-dependent histidine kinases whose autophosphorylation activity relies on ATP binding, ANP serves as a suitable surrogate ligand for evaluating the structural integrity and accessibility of ATP-binding sites. Similar ATP-analogue-based approaches have been widely employed for the structural characterization of histidine kinases and for the identification of ATP-competitive inhibitors targeting bacterial two-component systems [38,39,40].

The docking protocol validation criterion (RMSD < 2.0 Å) used in this study is consistent with established standards, as re-docking ANP into its original binding site, with a RMSD value of 1.54 Å, confirms the reliability of the docking approach. From a therapeutic perspective, the strong predicted binding of ANP to wild-type PhoQ, PhoP, and PmrB suggests that two-component system kinases may represent potential targets for ATP-competitive inhibition. However, these docking results are preliminary and require experimental validation, including ATPase inhibition assays, ITC/SPR binding studies, and structural approaches (X-ray/cryo-EM), followed by confirmation in biofilm and in vivo infection models to establish clinical relevance. 

Reduced ANP binding affinity observed in all mutant proteins indicates that amino acid substitutions likely alter key structural or catalytic regions, affecting ligand interaction and TCS signaling. The variable binding energy shifts across mutants further suggest that colistin resistance arises through protein-specific structural adaptations rather than a uniform mechanism.

## 5. Conclusions

This study provides molecular and structural insights into colistin resistance in *P. aeruginosa*. While most isolates remained susceptible to polymyxins, the detection of *mcr*-1 and the high prevalence of mutations in the *PhoPQ* and *PmrAB* regulatory systems indicate the presence of genetic mechanisms associated with resistance development. These findings suggest that continued reliance on polymyxins may promote the emergence of resistant strains if resistance determinants become more widespread. Structural analyses demonstrated that resistance-associated mutations alter protein–ligand interactions, supporting their potential role in modulating resistance-related signaling pathways. Together, these findings improve our understanding of the molecular basis of colistin resistance and provide a foundation for future studies aimed at validating potential therapeutic targets and monitoring the spread of resistance determinants in clinical settings.

## 6. Limitations of the Study

A key limitation of this study is the lack of associated clinical outcome data, including treatment response, mortality, and length of hospital stay. As a laboratory-based cross-sectional design, no longitudinal follow-up was performed to correlate resistance determinants with patient prognosis. Although the molecular and phenotypic findings provide insight into colistin resistance mechanisms in *Pseudomonas aeruginosa*, the clinical relevance of identified mutations and *mcr-1* carriage in relation to therapeutic efficacy remains unclear. Future prospective studies integrating standardized clinical outcomes are required to establish the translational and prognostic significance of these resistance markers.

## Figures and Tables

**Table 1:** Combined Distribution of Clinical Samples and *P. aeruginosa* Positive Isolates with Statistical Analysis.

Variable	Category	Total Samples *n* (%)	Positive *n* (%)
Gender	Male	1860 (58.33%)	240 (12.9)
	Female	1329 (41.67%)	144 (10.8)
Age Group (Years)	≤10	200 (6.27%)	17 (8.5)
	11–20	265 (8.31%)	29 (10.9)
	21–40	804 (25.21%)	96 (11.9)
	41–60	1068 (33.49%)	131 (12.3)
	>60	852 (26.71%)	111 (13.0)
Total		3189 (100%)	384 (12.0)

**Table 2:** Distribution of Positive Isolates by Specimen Source (*n* = 384).

Specimen Source	Positive Isolates (*n*)	Percentage (%)
Wound/Pus	146	38.0
Urine	104	27.1
Sputum	69	18.0
Blood	38	9.9
Others	27	7.0
Total	384	100

**Table 3:** Distribution of *P. aeruginosa* Isolates by Hospital Department (*n* = 384).

Hospital Department	Positive Isolates *n* (%)
Surgical wards	115 (30.0)
Intensive Care Unit (ICU)	96 (25.0)
Burn unit	69 (18.0)
Medical wards	58 (15.1)
Outpatient department (OPD)	31 (8.1)
Pediatric unit	15 (3.9)
Total	384 (100)

**Table 4:** Antibiotic Susceptibility Profile of *Pseudomonas aeruginosa* with Statistical Analysis (*n* = 384).

Antibiotic (Disc Potency)	Resistant *n* (%)
Piperacillin–Tazobactam (100/10 µg)	370 (96.4)
Ceftazidime (30 µg)	177 (46.2)
Cefepime (30 µg)	212 (55.2)
Imipenem (10 µg)	20 (5.2)
Meropenem (10 µg)	32 (8.4)
Doripenem (10 µg)	38 (10.0)
Cefoperazone–Sulbactam (75/30 µg)	319 (83.1)
Levofloxacin (5 µg)	211 (55.0)
Ciprofloxacin (5 µg)	187 (48.8)
Amikacin (30 µg)	27 (7.1)
Gentamicin (10 µg)	247 (64.2)
Tobramycin (10 µg)	232 (60.3)
Aztreonam (30 µg)	271 (70.6)
Polymyxin B (300 IU)	4 (1.0)
Colistin (MIC strip)	4 (1.0)

**Table 5:** Minimum Inhibitory Concentration (MIC) Profile of Selected Antibiotics Against *P. aeruginosa* (*n* = 384).

Antibiotic	MIC_50_ (µg/mL)	MIC_90_ (µg/mL)	MIC Range (µg/mL)
Colistin (Polymyxin E)	1	1	<0.25–2
Polymyxin B	1	1	<0.5–8
Meropenem	1	8	≤0.25–16
Imipenem	2	16	1–64

CLSI 2023 breakpoints (µg/mL): Colistin—S ≤ 2, R ≥ 4; Polymyxin B—S ≤ 2, R ≥ 4; Meropenem—S ≤ 2, I = 4, R ≥ 8; Imipenem—S ≤ 2, I = 4, R ≥ 8. Sensitive, Intermediate, and Resistance represent CLSI 2023 breakpoints (μg/mL).

**Table 6:** Association of Antibiotic-Resistant Genes with Phenotypic Resistance Profiles in *Pseudomonas aeruginosa*.

Isolate No.	Source	Detected Genes	Phenotypic Resistance Profile	OR	95% Cl	*p*-Value
21	Urine	*PhoP*, *PhoQ*, *PmrA*, *PmrB*	COL, PB, CAZ, FEP, CIP, SXT	2.3	1.0–5.6	0.052
43	Sputum	*mcr*-1, *PhoQ*, *PmrB*, *oprD*	COL, PB, MEM, IMP, CAZ, FEP, CIP	42.5	12.3–146.8	<0.001
78	Blood	*PhoP*, *PhoQ*, *PmrA*, *PmrB*, oprD	COL, PB, MEM, IMP, CAZ, FEP, CIP, TZP	2.8	1.2–6.5	0.015
119	Pus	*PhoP*, *PhoQ*, *PmrA*, *PmrB*, *mcr*-1	COL, PB, CAZ, FEP, CIP, SXT, AK	1.8	0.7–4.2	0.214
162	Wound swab	*oprD*, *PmrB*, *PhoQ*	MEM, IMP, CAZ, FEP, CIP	-	-	—

OR = odds ratio; CI = confidence interval. *p*-values are calculated using Fisher’s exact test. Statistically significant associations (*p* < 0.05) are in bold. — indicates that the OR could not be calculated due to zero values in the contingency table. Antibiotics: COL Colistin, PB Polymyxin B, CAZ Ceftazidime, FEP Cefepime, CIP Ciprofloxacin, SXT Sulfamethoxazole-Trimethoprim (co-trimoxazole), MEM Meropenem, IMP Imipenem, TZP Piperacillin–Tazobactam, AK Amikacin.

**Table 7:** Gene vs. Antibiotic Resistance Association Matrix in *Pseudomonas aeruginosa*.

Gene	COL	PB	MEM	IMP	CAZ	FEP	CIP	TZP	SXT	AK
*PhoP*	+	+	±	±	+	+	+	±	+	±
*PhoQ*	+	+	±	±	+	+	+	±	±	±
*PmrA*	+	+	±	±	+	+	±	±	±	±
*PmrB*	+	+	+	+	+	+	+	±	±	±
*mcr-1*	+++	+++	±	±	+	+	+	±	+	+
*oprD*	−	−	+++	+++	+	+	+	±	−	±

Note: +++ = strong association (high-confidence resistance link), + = moderate association, ± = variable/weak association, − = no known association. Antibiotics: COL Colistin, PB Polymyxin B, CAZ Ceftazidime, FEP Cefepime, CIP Ciprofloxacin, SXT Sulfamethoxazole-Trimethoprim (co-trimoxazole), MEM Meropenem, IMP Imipenem, TZP Piperacillin–Tazobactam, AK Amikacin.

**Table 8:** Classification of *P. aeruginosa* Isolates by Resistance Category with Regional Comparison.

Resistance Category	Current Study (*n* = 384)	Iraq (2026) [23]	Iran (2026) [24]	India (2025) [25]
Multidrug-Resistant (MDR)	363 (94.5%)	34(42.5%)	134 (41%)	69 (36.5%)
Extensively Drug-Resistant (XDR)	232 (60.4%)	22(27.5%)	82 (25%)	23 (12.0%)
Pan-Drug-Resistant (PDR)	0 (0%)	8(10%)	14 (4.4%)	7 (3.7%)
Sensitive	21 (5.5%)	16 (20%)	192 (59%)	120 (63.5%)
Total	384 (100%)	80 (100%)	326 (100%)	189 (100%)

Classification based on Magiorakos et al. (2012) [26]. Antimicrobial categories tested: Polymyxins (Colistin, Polymyxin B), Carbapenems (Imipenem, Meropenem, Doripenem), Cephalosporins (Ceftazidime, Cefepime), Penicillins (Piperacillin–Tazobactam, Cefoperazone–Sulbactam), Aminoglycosides (Amikacin, Gentamicin, Tobramycin), Fluoroquinolones (Ciprofloxacin, Levofloxacin), Monobactams (Aztreonam).

**Table 9:** Comparative Ligand–Receptor Interaction Profile Across All Target Proteins.

Protein System	Key Residues Involved	Interaction Types	No. of Interactions	Strongest Interaction (kcal/mol)	Key Binding Residue
Mut-PhoP	ARG179, SER135, ASP62, SER88, GLN183, PHE89	H-donor, H-acceptor, π–H	16	−4.3	ARG179
Wild-PhoP (PDB ID 2PKX)	LYS87, ARG111, THR100, GLU107, HIS104	H-donor, H-acceptor, Ionic	13	−9.1	LYS87/ARG111
Mut-mcr1	MET47, ALA15, THR79, GLY72	H-donor, H acceptor	10	−4.0	MET47
Wild mcr1-(PDB ID: 3PBT)	ILE347, ASN351, THR487, SER294, ALA488, ARG489	H-donor, H-acceptor, π–H	7	−3.4	ILE347
Mut-PhoQ	GLU271, ALA279, GLN281, LEU278, HIS286	H-donor, H-acceptor	6	−6.8	GLU271
Wild PhoQ (PDB ID: 1ID0)	LYS392, ARG431, ARG434, ARG439, GLN442, LEU446	H-donor, H-acceptor, π–H, π–cation	14	−12.0	LYS392
Mut-PmrB	ASN351, ASN355, ARG358, TYR359, GLY413	H-donor, H-acceptor, π–H, π–π	8	−2.0	ARG358
Wild-PmrB (PDB ID: 2JSO)	ASP37	H-donor	1	−9.8	ASP37

**Table 10:** Molecular docking results of wild-type and mutant forms of selected proteins (PhoP, PhoQ, PmrB, and mcr-1) showing docking score, RMSD (refine), and associated energy parameters (E_conf, E_place, E_score1, and E_refine) obtained using MOE.

Protein	Variant	Docking Score	Rmsd Refine	E_conf	E_place	E_score1	E_refine
PhoP	Mutant	−7.380	1.1953	−655.93	−100.76	−14.72	−46.64
	Wild	−6.807	3.1866	−1011.70	−73.74	−22.99	−61.709
PhoQ	Mutant	−7.787	2.0377	−648.93	−97.21	−11.77	−46.68
	Wild	−7.973	1.2971	−634.32	−115.18	−15.30	−49.24
PmrB	Mutant	−8.368	1.8461	−616.92	−108.27	−12.64	−40.53
	Wild	−6.545	1.4214	−658.79	−72.60	−10.87	−40.14
Mcr-1	Mutant	−6.979	2.0787	−636.59	−69.13	−11.45	−43.40
	Wild	−8.840	1.9965	−650.51	−117.50	−12.39	−42.64

## Data Availability

The datasets generated and/or analyzed during the current study are available from the corresponding author on reasonable request. Sequence data generated in this study have been analyzed using standard bioinformatics tools, and relevant data are included within the manuscript.

## Supplementary Materials

The following supporting information can be downloaded at: https://www.mdpi.com/article/10.3390/biom16070962/s1. Table S1. specific primers used for target antibiotics resistance genes. Table S2: Mutations identified in colistin resistance-associated genes among *P. aeruginosa* isolates (*n* = 384). Table S3. Comparative Ligand–Receptor Interaction Analysis of Mutant and Wild *PhoP.* Table S4. Comparative Ligand–Receptor Interaction Analysis of Mutant and Wild-Type *mcr*-1. Table S5. Comparative Ligand–Receptor Interaction Analysis of Mutant and Wild-Type-*PhoQ*. Table S6. Comparative Ligand–Receptor Interaction Analysis of Mutant and Wild-Type PmrB. Figure S1: (A: *P. aeruginosa* growth on MacConkey agar media showing pale or colorless colonies indicating non-lactose fermenter. (B) *P. aeruginosa* growth on cetrimide agar, colonies displayed greenish-blue pigmentation. (C): Gram staining of *P. aeruginosa* under microscope, revealed pink, rod-shaped. (D): API 20E biochemical profiling confirmed the identification of *P. aeruginosa* isolate 2212004 based on characteristic positive and negative enzymatic reactions. (E): Catalase tests were positive for *P. aeruginosa*. (F): oxidase tests were positive for *P. aeruginosa*. Figure S2: PCR amplification of the *oprL* gene yielded a 504 bp product, confirming the molecular identification of *Pseudomonas aeruginosa*. Figure S3: Comparative 2D interaction diagrams and 3D structural models mutant PhoP proteins. Figure S4: Comparative 2D interaction diagrams and 3D structural models Wild PhoP proteins. Figure S5: 2D and 3D-structure of Ligand–Receptor Interaction Analysis of mcr1 Mutant Protein. Figure S6: 2D and 3D-structure of Ligand–Receptor Interaction Analysis of mcr1 Wild Protein. Figure S7: 2D and 3D-structure of Ligand–Receptor Interaction Analysis Mutant Protein *PhoQ.* Figure S8: 2D and 3D-structure of Ligand–Receptor Interaction Analysis Wild Protein *PhoQ.* Figure S9: Comparative molecular docking interaction maps of mutant *PmrB* proteins, showing ligand–residue interactions including hydrogen bonding, π–π, and π–H interactions. Figure S10: Comparative molecular docking interaction maps of Wild *PmrB* proteins, showing ligand–residue interactions including hydrogen bonding, π–π, and π–H interactions.
