# Supplementary material for: In Silico Mutational Analysis of Two-Component System Genes Associated with Colistin Resistance in Clinical Pseudomonas aeruginosa Isolates from Peshawar
Source: Biomolecules. 2026 Jun 29;16(7):962. doi: 10.3390/biom16070962 (PMC13406812; doi:10.3390/biom16070962)
Supplement: Supplementary file 1 [file biomolecules-16-00962-s001.zip › biomolecules-4367141-supplementary.pdf]

biomolecules

Supplementary material

Table S1. specific primers used for target antibiotics resistance genes.

S.no	Target gene(s)	Primer sequence (5′-3′)	Product size (bp)	Annealing temperature (⁰C/Sec)	References
1	*oprL*	F:ATGGAAATGCTGAAATTCGGC<br>R: CTTCTTCAGCTCGACGCGACG	504	55/30	Current Study
2	*PhoP*	F: ATGCGCAGGTGATGATGAC<br>R: TCAGCGGTTGATGTAGGTGA	360	56/30	
3	*PhoQ*	F: ATCCGCTCCTGATCGTCTT<br>R CTTGATGTTGCCGTTGTTGC	405	59/30	
4	*PmrA*	F: GCTGACCTTCAACCTGAACA<br>R: TCGTCGTAGATCGCCTTGAT	344	56/30	
5	*PmrB*	F: ATCTGCTTGACGATCTGGTC<br>R: CGTGGTTGATGACGTTCTTC	421	57/30	
6	*mcr-1*	F: CGGTCAGTCCGTTTGTTC<br>R: CTTGGTCGGTCTGTAGGG	309	55/30	
7	*oprD*	F: ATGAACTGGGTCGTTCGCT<br>R: TCACTTGATGGTCGACGCC	504	58/30	

Table S2: Mutations identified in colistin resistance-associated genes among *P. aeruginosa* isolates (n = 384).

Gene	Isolates with Mutations n (%)	Mutations Identified	OR	95% CI	p-value
mcr-1	31 (8.0%)	p.S30, p.C54F	42.5	12.3–146.8	<0.001
pmrB	207 (54.0%)	p.S8X, p.G121D, p.Q196L, p.E400G, p.E461X	2.8	1.2–6.5	0.015
phoQ	169 (44.0%)	p.C28W, p.R42G, p.R51G, p.S63A, p.A88G, p.R173C, p.A192G, p.238W, p.A260T	2.1	0.9–5.1	0.078
phoP	138 (36.0%)	p.V208F, p.C260Y	2.3	1.0–5.6	0.052
pmrA	69 (18.0%)	p.S73, p.W145C, p.T167N, p.S187C, p.25W	1.5	0.5–4.2	0.412
oprD	336 (87.5%)	p.D236G	—	—	—

OR = odds ratio; CI = confidence interval. p-values calculated using Fisher's exact test. Statistically significant associations ($p < 0.05$) are in **bold**. — indicates OR could not be calculated due to zero values in the contingency table. Note: All isolates carried the intrinsic genes phoP, phoQ, pmrA, pmrB, and oprD. The percentages represent isolates with identified mutations in these genes, not gene presence. Only mcr-1 is a plasmid-acquired gene.

Table S3. Comparative Ligand–Receptor Interaction Analysis of Mutant and Wild PhoP

Protein	Ligand Atom	Receptor Atom	Residue	Interaction Type	Distance (Å)	Energy (kcal/mol)
**Mutant *PhoP***	O9	O	ARG179	H-donor	2.85	-4.3
	O15	O	SER135	H-donor	3.04	-3.2
	O27	OG	SER135	H-donor	2.75–2.86	-1.5 to -1.4
	O31	OD1	ASP62	H-donor	2.88–2.92	-1.9 to -2.3
	N41	OG	SER88	H-donor	3.13–3.23	-1.2 to -1.6
	O2	NE2	GLN183	H-acceptor	2.87–3.05	-4.0 to -3.6
	O14	NE2	GLN183	H-acceptor	3.41–3.48	-1.1 to -1.2

	6-ring	CD1	PHE89	π–H interaction	4.45–4.49	-0.8 to -0.9
**Wild-*PhoP* (PDB ID: 2PKX)**	N8	OG1	THR100 (B)	H-donor	3.29	-2.3
	C17	OE1	GLU107 (A)	H-donor	2.92	-0.5
	O2	NZ	LYS87 (B)	H-acceptor	3.24	-0.5
	O6	NZ	LYS87 (B)	H-acceptor	2.95	-9.1
	O7	NH2	ARG111 (B)	H-acceptor	2.48	-2.9
	O20	ND1	HIS104 (A)	H-acceptor	3.51	-1.1
	O2	NZ	LYS87 (B)	Ionic	3.24	-3.1
	O3	NZ	LYS87 (A)	Ionic	3.15	-3.5
	O6	NZ	LYS87 (B)	Ionic	2.95	-4.8
	O7	NH2	ARG111 (B)	Ionic	2.48	-9.0
	O10	NH2	ARG111 (A)	Ionic	3.34	-2.6
	O10	NZ	LYS87 (B)	Ionic	3.36	-2.5
	O11	NH2	ARG111 (A)	Ionic	3.95	-0.6

Table S4. Comparative Ligand–Receptor Interaction Analysis of Mutant and Wild-Type *mcr-1*.

Protein	Ligand Atom	Receptor Atom	Residue	Interaction Type	Distance (Å)	Energy (kcal/mol)
Mutant *mcr-*1	O5	SD	MET47	H-donor	3.13–3.27	-4.0 to -3.7
	N11	SD	MET47	H-donor	3.49–3.53	-2.7
	O31	O	ALA15	H-donor	3.00	-2.7
	C33	OG1	THR79	H-donor	3.22–3.34	-0.9 to -0.7
	O14	CA	GLY72	H-acceptor	3.49	-0.6
Wild *mcr*-1 (3PBT)	O3	O	ILE347 (A)	H-donor	2.87	-3.4
	O31	OD1	ASN351 (A)	H-donor	2.60	-2.6
	O14	OG1	THR487 (A)	H-acceptor	3.19	-1.0
	O24	OG	SER294 (A)	H-acceptor	2.90	-0.8
	6-ring	CA	ALA488 (A)	π–H interaction	3.91	-0.5
	5-ring	CA	ALA488 (A)	π–H interaction	4.09	-0.5
	5-ring	N	ARG489 (A)	π–H interaction	4.09	-0.5

Table S5. Comparative Ligand–Receptor Interaction Analysis of Mutant and Wild-Type-*PhoQ*.

Protein	Ligand Atom	Receptor Atom	Residue	Interaction Type	Distance (Å)	Energy (kcal/mol)
Mutant PhoQ	O3	OE1	GLU271	H-donor	2.82	-6.8
	C25	O	ALA279	H-donor	3.28	-0.7
	O27	O	GLN281	H-donor	3.08	-2.4
	O14	CA	LEU278	H-acceptor	3.35	-0.5
	O14	N	ALA279	H-acceptor	2.86	-3.6
	O18	NE2	HIS286	H-acceptor	2.74	-1.4
	O3	OE1	GLN442 (A)	H-donor	3.22	-2.0

https://doi.org/10.3390/biom16070962

Wild PhoQ (PDB ID: 1ID0)	O9	OD1	ASN385 (A)	H-donor	2.77	-2.0
	N11	OD1	ASN389 (A)	H-donor	3.08	-1.4
	O31	O	ASP430 (A)	H-donor	2.71	-1.7
	N41	O	VAL444 (A)	H-donor	2.87	-3.5
	O2	NZ	LYS392 (A)	H-acceptor	2.69	-12.0
	O2	OH	TYR393 (A)	H-acceptor	2.76	-1.1
	O3	NH2	ARG439 (A)	H-acceptor	2.80	-0.6
	O3	NE2	GLN442 (A)	H-acceptor	2.72	-1.2
	O8	NH2	ARG434 (A)	H-acceptor	3.13	-3.2
	O14	N	LEU446 (A)	H-acceptor	2.82	-4.7
	N47	N	GLY432 (A)	H-acceptor	2.77	-2.7
	6-ring	CD	ARG431 (A)	π–H interaction	4.14	-1.3
	6-ring	NH1	ARG434 (A)	π–cation interaction	4.14	-1.2

Table S6. Comparative Ligand–Receptor Interaction Analysis of Mutant and Wild-Type PmrB.

**Protein**	**Ligand A.**	**Receptor Atom**	**Residue**	**Interaction Type**	**Distance (Å)**	**Energy (kcal/mol)**
Mutant PmrB	O9	O	ASN351	H-donor	3.38	-1.3
	C19	OD1	ASN355	H-donor	2.91	-1.2
	O8	CA	GLY415	H-acceptor	3.21	-1.1
	O9	NH1	ARG358	H-acceptor	2.65	-1.5
	O14	N	GLY413	H-acceptor	3.32	-2.0
	O17	NH1	ARG358	H-acceptor	2.72	-0.6
	5-ring	ND2	ASN355	π–H interaction	3.43	-0.6
	6-ring	6-ring	TYR359	π–π interaction	3.93	~0.0
Wild-PmrB (2JSO)	O3	OD2	ASP37 (A)	H-donor	2.87	-9.8

https://doi.org/10.3390/biom16070962

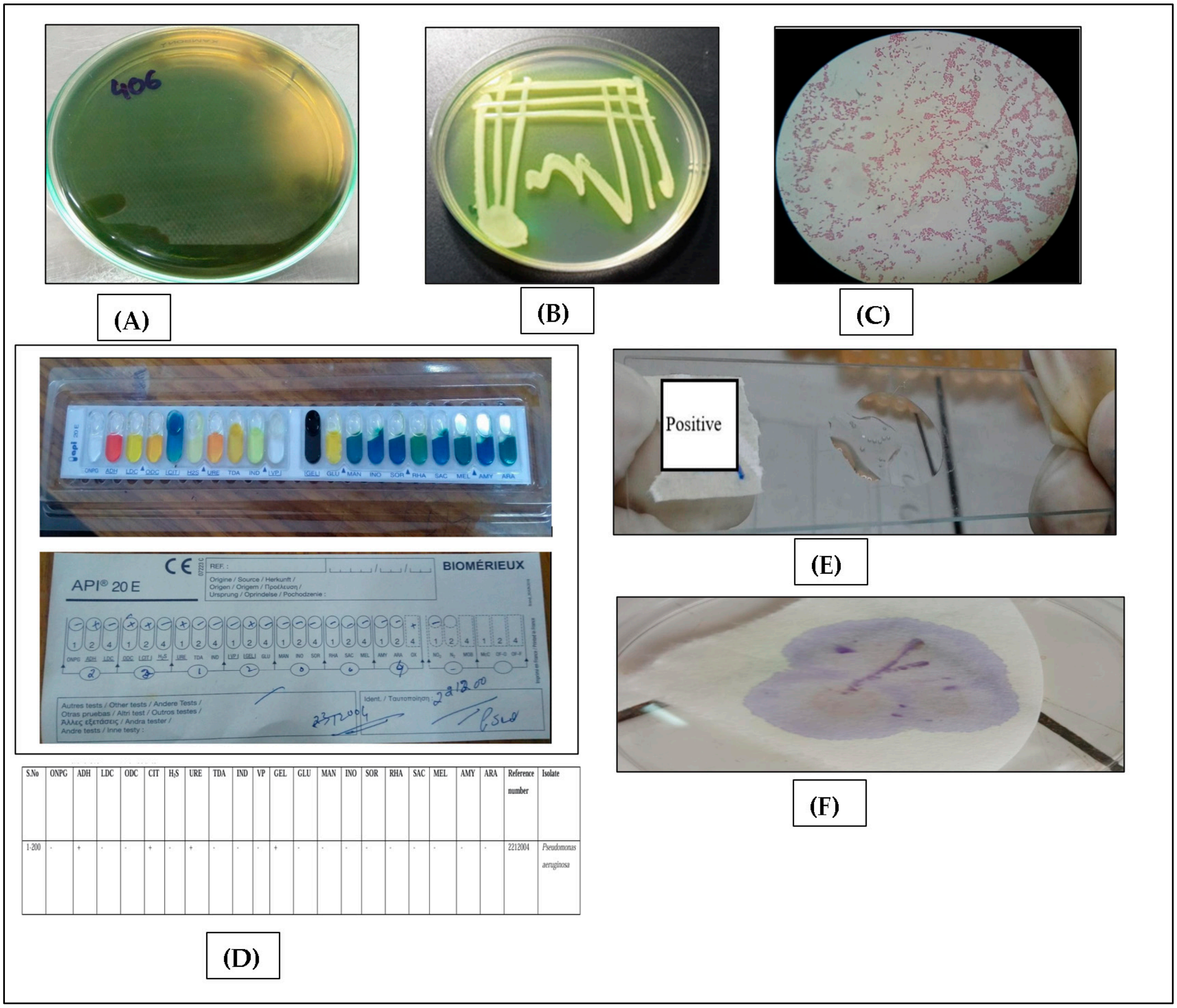

S.No	ONPG	ADH	LDC	ODC	CIT	H_2S	URE	TDA	IND	VP	GEL	GLU	MAN	INO	SOR	RHA	SAC	MEL	AMY	ARA	Reference number	Isolate
1-200	-	+	-	-	+	-	+	-	-	-	+	-	-	-	-	-	-	-	-	-	2212004	*Pseudomonas aeruginosa*

Figure S1: **(A:** *P. aeruginosa* growth on MacConkey agar media showing pale or colorless colonies indicating non-lactose fermenter. **(B)** *P. aeruginosa* growth on cetrimide agar, colonies displayed greenish-blue pigmentation. **(C):** Gram staining of *P. aeruginosa* under microscope, revealed pink, rod-shaped. **(D):** API 20E biochemical profiling confirmed the identification of *P. aeruginosa* isolate 2212004 based on characteristic positive and negative enzymatic reactions. **(E):** Catalase tests were positive for *P. aeruginosa*. **(F):** oxidase tests were positive for *P. aeruginosa*.

https://doi.org/10.3390/biom16070962

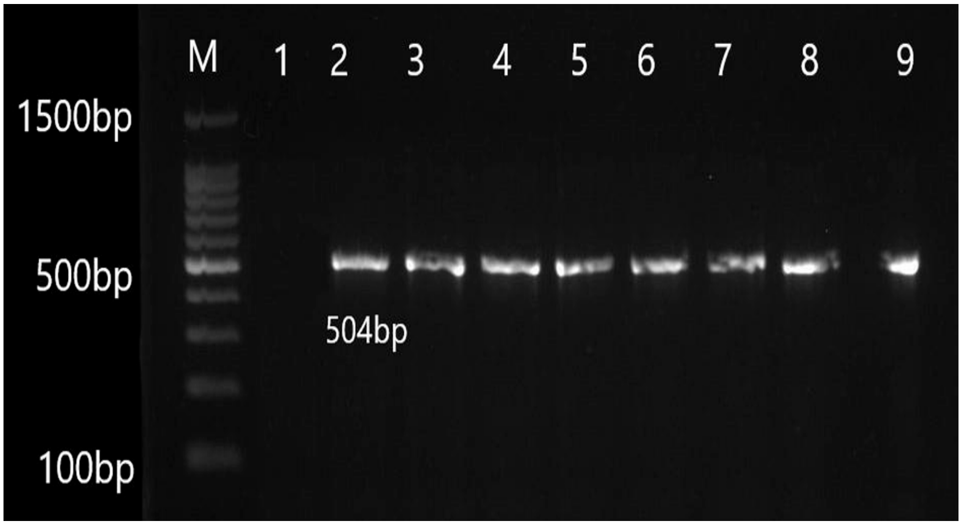

Figure S2: PCR amplification of the *oprL* gene yielded a 504 bp product, confirming the molecular identification of *Pseudomonas aeruginosa*.

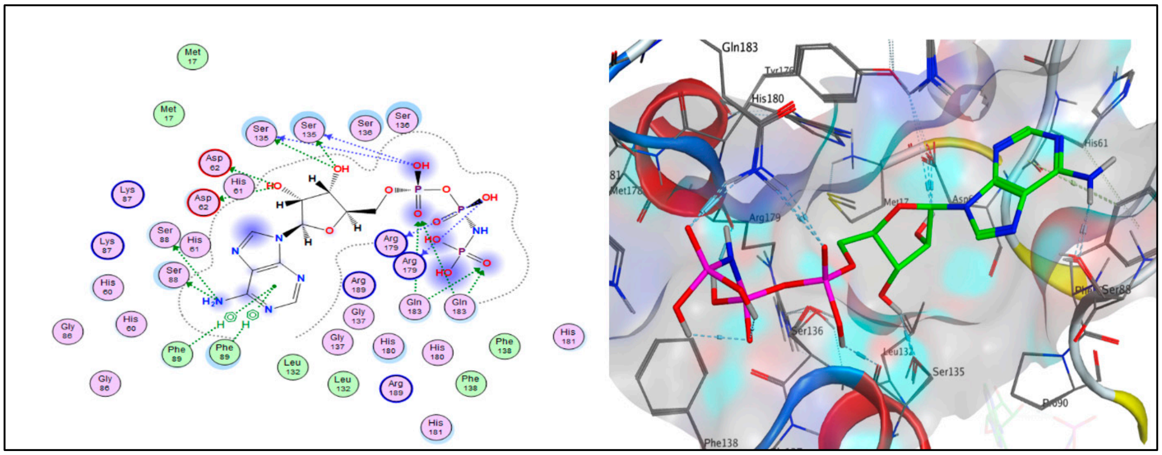

Figure S3: Comparative 2D interaction diagrams and 3D structural models mutant PhoP proteins.

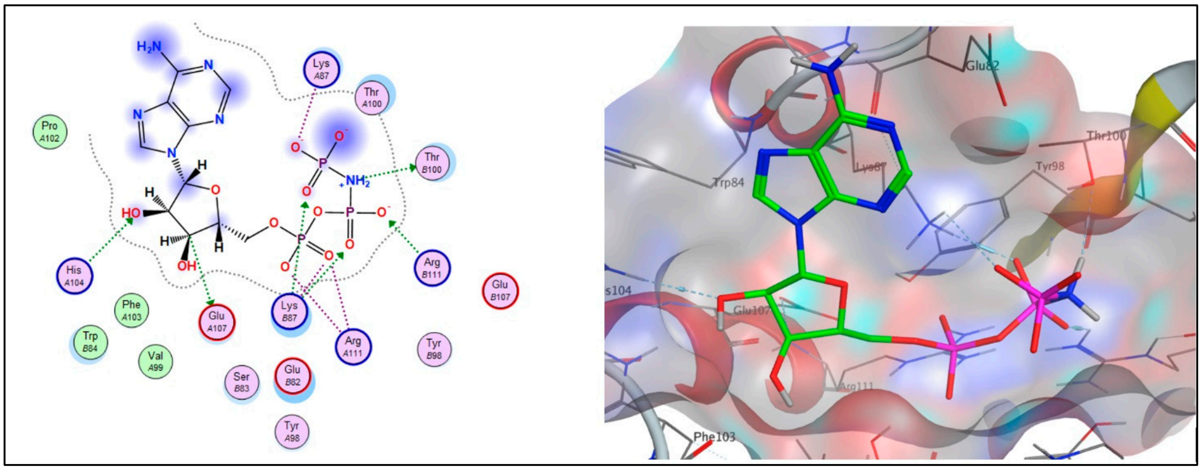

Figure S4: Comparative 2D interaction diagrams and 3D structural models Wild PhoP proteins.

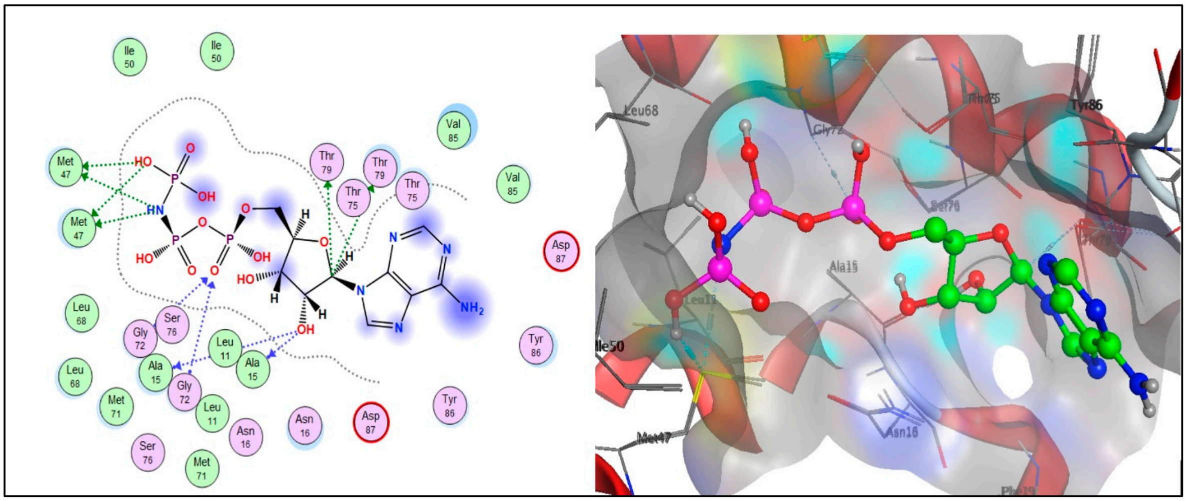

Figure S5: 2D and 3D-structure of Ligand–Receptor Interaction Analysis of mcr1 Mutant Protein

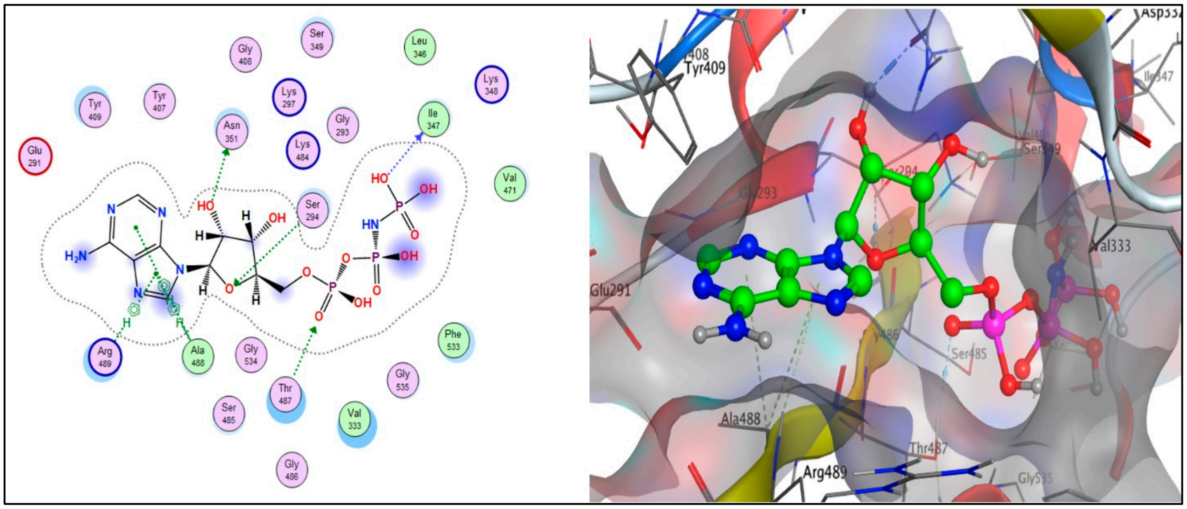

Figure S6: 2D and 3D-structure of Ligand–Receptor Interaction Analysis of mcr1 Wild Protein

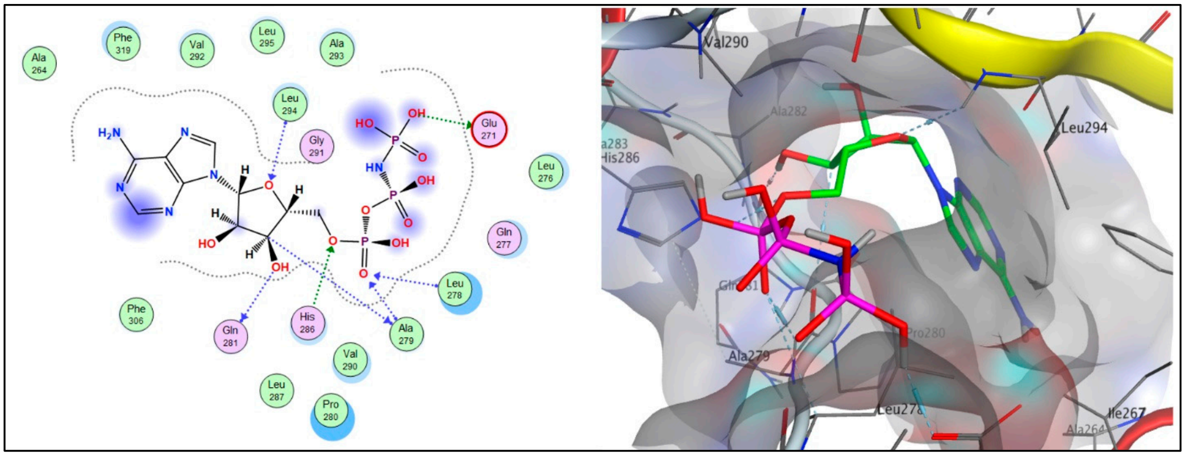

Figure S7: 2D and 3D-structure of Ligand–Receptor Interaction Analysis Mutant Protein *PhoQ*

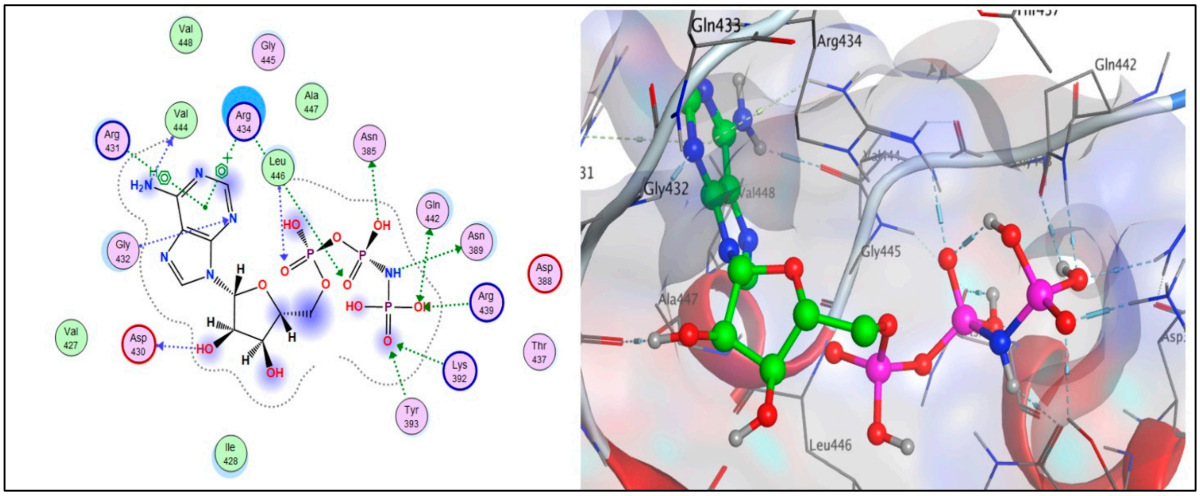

Figure S8: 2D and 3D-structure of Ligand–Receptor Interaction Analysis Wild Protein *PhoQ*

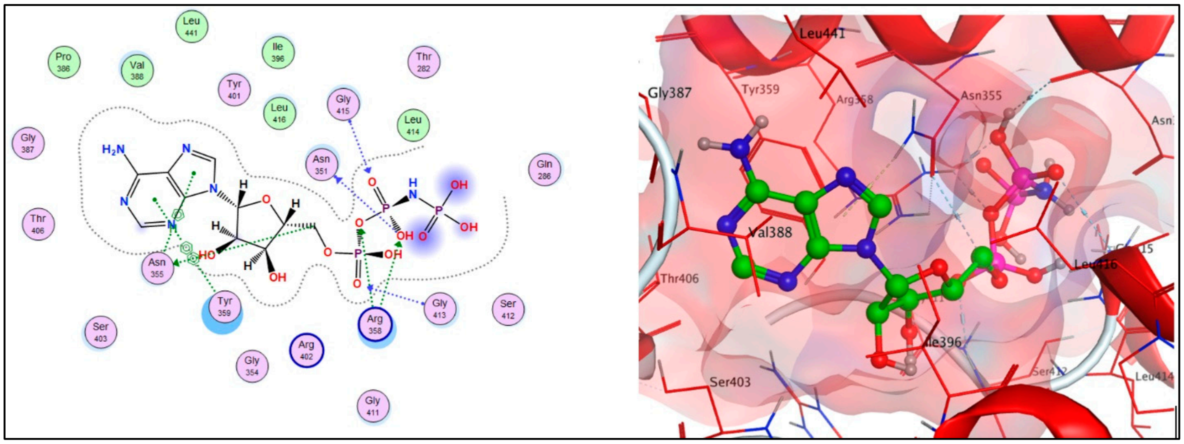

Figure S9: Comparative molecular docking interaction maps of mutant *PmrB* proteins, showing ligand–residue interactions including hydrogen bonding, π–π, and π–H interactions

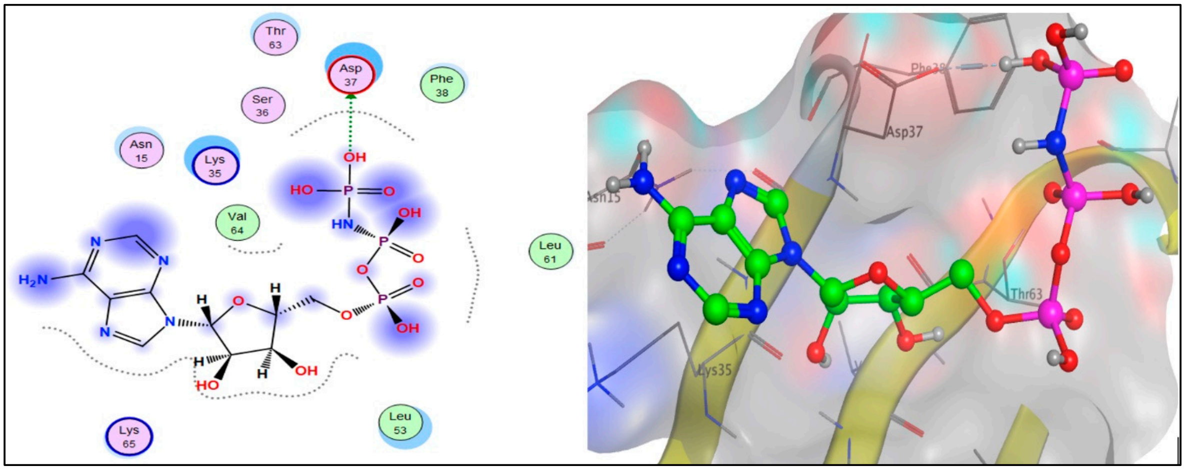

Figure S10: Comparative molecular docking interaction maps of Wild *PmrB* proteins, showing ligand–residue interactions including hydrogen bonding, π–π, and π–H interactions

https://doi.org/10.3390/biom16070962

Disclaimer/Publisher's Note: The statements, opinions and data contained in all publications are solely those of the individual author(s) and contributor(s) and not of MDPI and/or the editor(s). MDPI and/or the editor(s) disclaim responsibility for any injury to people or property resulting from any ideas, methods, instructions or products referred to in the content.

https://doi.org/10.3390/biom16070962
